# Comparative genomic analysis of *Mycobacterium tuberculosis* clinical isolates

**DOI:** 10.1186/1471-2164-15-469

**Published:** 2014-06-13

**Authors:** Fei Liu, Yongfei Hu, Qi Wang, Hong Min Li, George F Gao, Cui Hua Liu, Baoli Zhu

**Affiliations:** CAS key Laboratory of Pathogenic Microbiology and Immunology, Institute of Microbiology, Chinese Academy of Sciences, Beijing, China; Institute for Tuberculosis Research, the 309th Hospital, Beijing, China

**Keywords:** *Mycobacterium tuberculosis*, Drug resistance, Single nucleotide polymorphisms, Whole genome sequencing, Evolution

## Abstract

**Background:**

Due to excessive antibiotic use, drug-resistant *Mycobacterium tuberculosis* has become a serious public health threat and a major obstacle to disease control in many countries. To better understand the evolution of drug-resistant *M. tuberculosis* strains, we performed whole genome sequencing for 7 *M. tuberculosis* clinical isolates with different antibiotic resistance profiles and conducted comparative genomic analysis of gene variations among them.

**Results:**

We observed that all 7 *M. tuberculosis* clinical isolates with different levels of drug resistance harbored similar numbers of SNPs, ranging from 1409–1464. The numbers of insertion/deletions (Indels) identified in the 7 isolates were also similar, ranging from 56 to 101. A total of 39 types of mutations were identified in drug resistance-associated loci, including 14 previously reported ones and 25 newly identified ones. Sixteen of the identified large Indels spanned PE-PPE-PGRS genes, which represents a major source of antigenic variability. Aside from SNPs and Indels, a CRISPR locus with varied spacers was observed in all 7 clinical isolates, suggesting that they might play an important role in plasticity of the *M. tuberculosis* genome. The nucleotide diversity (Л value) and selection intensity (dN/dS value) of the whole genome sequences of the 7 isolates were similar. The dN/dS values were less than 1 for all 7 isolates (range from 0.608885 to 0.637365), supporting the notion that *M. tuberculosis* genomes undergo purifying selection. The Л values and dN/dS values were comparable between drug-susceptible and drug-resistant strains.

**Conclusions:**

In this study, we show that clinical *M. tuberculosis* isolates exhibit distinct variations in terms of the distribution of SNP, Indels, CRISPR-cas locus, as well as the nucleotide diversity and selection intensity, but there are no generalizable differences between drug-susceptible and drug-resistant isolates on the genomic scale. Our study provides evidence strengthening the notion that the evolution of drug resistance among clinical *M. tuberculosis* isolates is clearly a complex and diversified process.

**Electronic supplementary material:**

The online version of this article (doi: 10.1186/1471-2164-15-469) contains supplementary material, which is available to authorized users.

## Background

The emergence and transmission of drug-resistant *M. tuberculosis* strains, especially Multidrug-resistant (MDR) and extensively drug-resistant (XDR) strains pose significant clinical, economic, as well as societal challenges. According to WHO report, there were an estimated 8.6 million incident cases of TB worldwide in 2012. Most of the estimated number of cases in 2012 occurred in Asia (58%) and the African Region (27%). The five countries with the largest number of incident cases include: India (2.0–2.4 million, in 2012), China (0.9–1.1 million, in 2012), South Africa (0.4–0.6 million, in 2012), Indonesia (0.4–0.5 million, in 2012) and Pakistan (0.3–0.5 million, in 2012). India and China alone accounted for 26% and 12% of global cases, respectively. In addition, the global estimate of the burden of MDR-TB was 300, 000 cases among notified TB patients in 2012. India and China were the two countries estimated to have the largest numbers of MDR-TB patients (both over 50,000) [1]. The latest nationwide baseline survey for TB drug resistance carried out in China for the 2007 and 2008 reported that 8.32% of pulmonary TB patients in China suffered from MDR-TB and 0.68% from XDR-TB. In 2007, there were an estimated 110,000 incident cases of MDR-TB and 8,200 incident cases of XDR-TB [2]. Furthermore, most cases of MDR- and XDR-TB were shown to be the result of primary transmission, suggesting that many of the new TB cases suffer from the most intractable types of highly drug-resistant *M. tuberculosis* strains [2, 3]. Antibiotic susceptibility profiles and the corresponding resistance determinants of *M. tuberculosis* have been extensively reported. However, the genome variations and evolution of drug resistance in *M. tuberculosis* are still not well explained. Determining the genome components and variations within natural populations of *M. tuberculosi*s isolates with different antibiotic susceptibility profiles may provide a novel perspective on the evolution of drug resistance in *M. tuberculo*sis and enable us to better understand and control drug-resistant TB.

The *Mycobacterium tuberculosis* complex (MTBC) lineages were considered to be monomorphic, but more and more studies have confirmed the extensive genetic diversity and genome plasticity of the mycobacterial genome through molecular typing techniques such as IS6110-RFLP, spoligotyping, and MIRU-VNTR [4–6]. With the advent of high throughput Next Generation Sequencing technologies (NGS), multiple genome sequences from different strains of a single species can provide comprehensive information for exploring the relationship between genotypes and phenotypes with unprecedented resolution. In this study, we used the Illumina GAIIx sequencing platform to generate a high-quality and annotated draft genome for 7 *M. tuberculosis* clinical isolates with different antibiotic resistance phenotypes in order to better understand the evolution of drug resistance in *M. tuberculosis* isolates in a clinical context. Comparative genomic analyses of these 7 strains as well as 7 other previously published *M. tuberculosis* genomes have revealed some genomic variations which might underlie diverse phenotypes among those strains, but no generalizable differences were identified between drug-susceptible and drug-resistant isolates on the genomic scale. Our study adds some new knowledge on genomic variability and evolution of drug-resistant *M. tuberculosis*.

## Results

### Whole genome sequencing statistics

The detailed epidemiologic and clinical data of the selected *M. tuberculosis* isolates were summarized in Table 1. The basic whole genome sequencing statistics are shown in Additional file 1: Table S1. The coverage ranged between 200× and 560×, and the completion was 97.43-97.82%. By comparing the sequenced *M. tuberculosis* clinical isolates to H37Rv, we observed that all 7 isolates with different levels and profiles of drug resistance harbored similar numbers of SNPs, ranging from 1409–1464. The numbers of insertion/deletions (Indels) identified in the 7 isolates were also similar, ranging from 56 to 101.

**Table 1:** **Epidemiologic and clinical data of clinical** ***M. tuberculosis*** **isolates**

Isolates	Type	Drug resistance profiles^a^	Age, years	Gender	Geographic location	Year of isolation	Treatment history	Clinical outcome	24 locus MIRU-VNTR profiles
Mtb562	Susceptible	None	22	Male	Liaoning	2010	New	Cure	223224163533-454334682431
Mtb526	MDR	INH, RMP, STR	39	Male	Shanxi	2011	Retreated	Cure	233224163533-454344672432
Mtb194	Pre-XDR	INH, RMP, STR, EMB, OFX, LVX	21	Female	Beijing	2010	New	Cure	213224163433-243344572422
Mtb293	Pre-XDR	INH, RMP, OFX, LVX, PAS, ETH	35	Female	Heilongjiang	2009	n.a.	n.a.	233324163523-454344682432
Mtb940	Pre-XDR	INH, RMP, STR, EMB, PAS, OFX, LVX, ETH	63	Male	Hebei	2010	New	Cure	233324143533-254344672432
Mtb984	XDR	INH, RMP, STR, EMB, OFX, LVX, KAN	72	Male	Anhui	2011	Retreated	Cure	233424173534-254344482432
Mtb43	XDR	INH, RMP, STR, EMB, PZA, OFX, LVX, KAN, CAP, AMK, PAS, ETH	47	Male	Henan	2009	Retreated	Died	232224153433-454344582432

*n.a.* = not available.

^a^INH, isoniazid; RMP, rifampicin; STR, streptomycin; EMB, ethambutol; PZA, pyrazinamide; OFX, ofloxacin; LVX, levofloxacin; KAN, kanamycin; CAP, capreomycin; AMK, amikacin; PAS, para-amino salicylic acid; ETH, ethionamide.

### SNP clustering and distribution in the *M. tuberculosis* genomes

**Figure 1:**
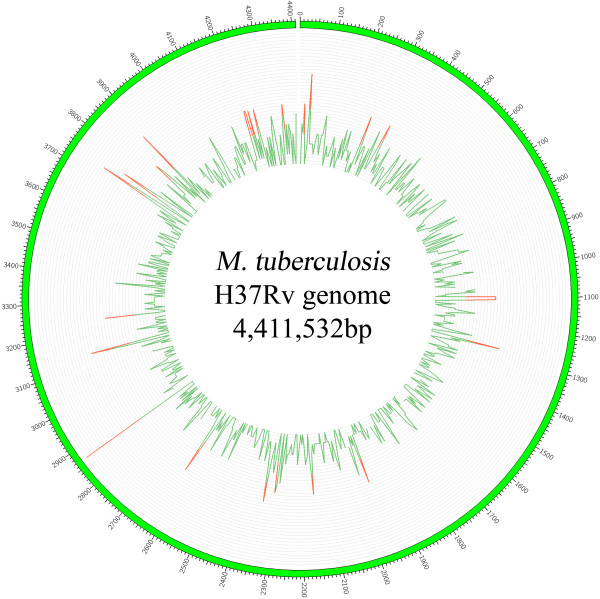
**SNP density map constructed using Circos.** Note: Green bars means the density of SNPs in non-overlapping 5-kb regions; Red bars means the regions with significantly high SNP density.

Further comparative genomic analysis identified a total of 1871 non repetitive SNPs, among which a common pool of 1102 SNPs were shared by the 7 isolates. More detailed information on total SNPs as well as SNPs in each isolate relative to H37Rv are summarized in Additional file 2: Table S2. To identify regions of SNP clustering, SNP density was estimated throughout the genomes using a sliding window of 5 kb. The resulting SNP density map shows a non-random distribution of SNPs, with 25 regions having statistically significant clusters (red bars in Figure 1). The detailed information on the 25 regions with significantly high SNP density is shown in Additional file 2: Table S2. We further analyzed the distribution of SNPs according to the different classes of the Clusters of Orthologous Groups (COG) [7–9]. We found that SNPs were significantly under-represented in genes belonging to secondary metabolites biosynthesis, transport, and catabolism (class Q), while genes whose functions were unknown (class S) were significantly enriched in SNPs (p < 0.01) (Additional file 3: Figure S1). SNPs were also slightly over-represented in genes belonging to several other classes such as class M (Cell wall/membrane/envelope biogenesis), class R (General function), class V (Defense mechanisms), class J (Translation, ribosomal structure and biogenesis), class K (Transcription), class T (Signal transduction mechanisms), and class N (Cell motility).

### Genomic insertions and deletions

We further analyzed large insertions and deletions (designated as those insertions or deletions are of 20 base pair long or above) in clinical *M. tuberculosis* relative to *M. tuberculosis* H37Rv. In total, 1 non strain-specific and 29 strain-specific large insertions as well as 2 non strain-specific and 61 strain-specific large deletions were identified. Sixteen of those Indels spanned PE-PPE-PGRS genes, which have been considered a major source of antigenic variability [10]. Many Indels were identified both in drug-susceptible and drug-resistant strains.

### CRISPR distribution in the *M. tuberculosis* genomes

CRISPRfinder was used to identify putative CRISPR loci in the genomes of the 7 *M. tuberculosis* isolates. In contrast to the *M. tuberculosis* lab strain H37Rv, which was predicted to have two CRISPR loci, all the 7 clinical *M. tuberculosis* isolates sequenced in this study as well as two other previously sequenced clinical *M. tuberculosis* isolates (including CCDC5079 and CCDC5180) were predicted to have only one of the two CRISPR loci. While the spacers in the CRISPR were identical among 5 clinical isolates including CCDC5079, CCDC5180 and three of our clinical isolates (Mtb562, Mtb 526 and Mtb43), other isolates had high variability in the spacers (Additional file 4: Figure S2). No correlation between antibiotic resistance and the presence of CRISPR-cas locus was observed.

### Gene mutations associated with drug resistance in M. tuberculosis

The detailed information on mutations identified in drug resistance-associated loci of the 7 Chinese clinical isolates is summarized in Table 2 and 3. A total of 39 types of mutations were identified in drug resistance-associated loci, including 14 previously reported ones and 25 newly identified ones. The levels of correlation between phenotypic drug resistance and drug resistance-associated mutations varied greatly for different drugs, ranging from 0% (for para-aminosalicylic acid and ethionamide) to 100% (isoniazid). We also identified 20 known or putative drug efflux pumps with non-synonymous SNPs in MDR, pre-XDR and XDR *M. tuberculosis* isolates but not in H37Rv strain (Additional file 5: Table S3). We further over expressed the mutated drug efflux pump genes in the drug-susceptible reference H37Rv strain and determined MICs of those recombinant strains. No increased drug resistance was observed for all examined strains over expressing mutated drug efflux pump genes. We also performed genetic studies by creating point mutations in the susceptible reference strain H37Rv using the pJV53K system for some other potential drug resistance-associated mutations identified in this study [11], but also could not confirm their function in causing drug resistance (data not shown).

**Table 2:** **SNPs and Indels identified in antibiotic resistance-associated regions in** ***M. tuberculosis*** **isolates**

			Mutations in target gene or intergenic regions (corresponding drugs)^a^						
Isolates	Rv1483( ***mabA*** )^b^ (INH)	Rv1484 ( ***inhA*** ) (INH)	Rv1592c (INH)	Rv1908c ( ***katG*** ) (INH)	Rv2247 ( ***accD6*** ) (INH)	Rv2428 ( ***ahpC*** ) (INH)	Rv2846c ( ***efpA*** ) (INH)	Rv0667 ( ***rpoB*** )^c^ (RMP)	Rv0682 ( ***rpsL*** ) (STR)
Mtb562	None	None	T70(del),E321^e,f^,I322V^f,g^	None	None	None	None	A1075^e,f^	None
Mtb526	None	None	T70(del),E321^e,f^,I322V^f,g^	S315T^d,g^,R463L^g^	D200^e,f^,D229G^g^	S40N^f,g^	None	L511P^d,g^,A1075^e,f^	K43R^d,g^,K121^e,f^
Mtb194	None	None	T70(del),E321^e,f^,I322V^f,g^	R463L^g^	D200^e,f^,D229G^g^	None	None	A1075^e,f^	K121^e,f^
Mtb293	None	None	T70(del),E321^e,f^,I322V^f,g^	C171G^f,g^,R463L^g^	D200^e,f^,D229G^g^	None	None	A1075^e,f^	K121^e,^
Mtb940	None	None	T70(del),E321^e,f^,I322V^f,g^	R463L^g^	D200^e,f^,D229G^g^	None	None	A1075^e,f^	K121^e,f^
Mtb984	None	G3 ^e,f^	T70(del),E321^e,f^,I322V^f,g^	S315T^d,g^,R463L^g^	D200^e,f^,D229G^g^	None	F128^e,f^	L511P^d,g^,S512G^g^,D516G^g^,A1075^e,f^	K43R^d,g^,K121^e,f^
Mtb43	T-8C^d^	G3 ^e,f^	T70(del),E321^e,f^,I322V^f,g^	S315T^d,g^,R463L^g^	D200^e,f^,D229G^g^	None	None	S531L^d,g^	K43R^d,g^,K121^e,f^

^a^“R”, resistance of isolates to the corresponding anti-TB drug; “S”, sensitivity of isolates to the corresponding anti-TB drug; “del”, deletion; INH, isoniazid; RMP, rifampicin; STR, streptomycin; EMB, ethambutol; PZA, pyrazinamide; OFX, ofloxacin; LVX, levofloxacin; KAN, kanamycin; CAP, capreomycin; AMK, amikacin; ETH, ethionamide.

^b^intergenic regions.

^c^nucleotide mutational position is relative to *Mycobacterium tuberculosis* H37Rv *rpoB*, and amino acid position is relative to *Escherichia coli* numbering.

^d^drug resistance-asssociated mutations with high confidence.

^e^synonymous.

^f^newly identified mutations.

^g^non-synonymous.

**Table 3:** **SNPs and identified in antibiotic resistance-associated regions in** ***M. tuberculosis*** **isolate** ***s***

			Mutations in target gene or intergenic regions (corresponding drugs)^a^						
Isolates	Rv3919c ( ***gidB*** ) (STR)	Rv3793 ( ***embC*** ) (EMB)	Rv3794 ( ***embA*** ) (EMB)	Rv3795 ( ***embB*** ) (EMB)	Rv2043c ( ***pncA*** ) (PZA)	Rv0006 ( ***gyrA*** ) (OFX, LVX)	Rvnr01 ( ***rrs*** ) (KAN, CAP, AMK)	Rv1694 ( ***tlyA*** ) (KAN, CAP, AMK)	Rv3854c ( ***ethA*** ) (ETH)
Mtb562	S100F^g^	R927^e^	None	None	None	S95T^g^	None	None	Q360H^g^
Mtb526	E92D^g^,S100F^g^,A205^e^	V885M^f,g^,R927^e^	C76^e^	None	None	E21Q^g^,S95T^g^,G668D^f,g^	None	L11^e^	Q360H^g^
Mtb194	E92D^g^,S100F^g^,A205^e^	V885M^f,g^,R927^e^	C76^e^	None	None	E21Q^g^,S95T^g^,G668D^f,g^	None	L11^e^	Q360H^g^
Mtb293	E92D^g^,S100F^g^,A205^e^	V885M^f,g^,R927^e^	C76^e^	None	None	E21Q^g^,S95T^g^,G668D^f,g^	None	L11^e^	Q360H^g^
Mtb940	E92D^g^,S100F^g^,A205^e^	V885M^f,g^,R927^e^	C76^e^	None	None	E21Q^g^,S95T^g^,G668D^f,g^	None	L11^e^	Q360H^g^
Mtb984	E92D^g^,S100F^g^,A205^e^	V885M^f,g^,R927^e^	G5S^f,g^,C76^e^	G406S^d,g^	F94S^f,g^	E21Q^g^,D94G^d,g^,S95T^g^, G668D^f,g^	None	L11^e^	Q360H^g^
Mtb43	E92D^g^,S100F^g^,A205^e^	V885M^f,g^	C76^e^	M306V^d,g^	T76I^g^	E21Q^g^,D94G^d,g^,S95T^g^,G668D^f,g^	G1332A,A1401G	L11^e^	P164L^f,g^,Q360H^g^

^a^“R”, resistance of isolates to the corresponding anti-TB drug; “S”, sensitivity of isolates to the corresponding anti-TB drug; “del”, deletion; INH, isoniazid; RMP, rifampicin; STR, streptomycin; EMB, ethambutol; PZA, pyrazinamide; OFX, ofloxacin; LVX, levofloxacin; KAN, kanamycin; CAP, capreomycin; AMK, amikacin; ETH, ethionamide.

^b^intergenic regions.

^c^nucleotide mutational position is relative to *Mycobacterium tuberculosis* H37Rv *rpoB*, and amino acid position is relative to *Escherichia coli* numbering.

^d^drug resistance-asssociated mutations with high confidence.

^e^synonymous.

^f^newly identified mutations.

^g^non-synonymous.

### Genetic diversity and selection intensity in the *M. tuberculosis* genomes

We used the whole genome sequences of the *M. tuberculosis* isolates for genetic diversity and selection intensity analysis and the data were shown in Additional file 6: Table S4. The nucleotide diversity (Л value) for the whole genome sequences of the 7 newly sequenced clinical isolates were similar, ranging from 0.00033 to 0.00036. There was no significant differences in Л values between drug-susceptible isolates and drug-resistant isolates (0.00024 versus 0.00021), while the Л value was significantly higher among clinical isolates (0.00033) as compared with lab strains (0.00004). The dN/dS values for the whole genome sequences were similar among isolates with different drug resistance profiles, ranging among 0.608885 to 0.637365. There was no significant differences in dN/dS values between drug-susceptible isolates and drug-resistant isolates (0.66891 versus 0.687259), while the dN/dS value was significantly lower among clinical isolates (0.66018) as compared with lab strains (0.765664). We observed significant differences in Л values between our 7 clinical isolates and 5 previously described clinical isolates (0.00008 versus 0.00057). But when we analyzed our 7 isolates together with the two Beijing lineage strains (CCDC5079 and CCDC5080) from the 5 previously described clinical isolates, the Л value increased from 0.00008 to 0.00028.

### Phylogenetic analysis of *M. tuberculosis* isolates

**Figure 2:**
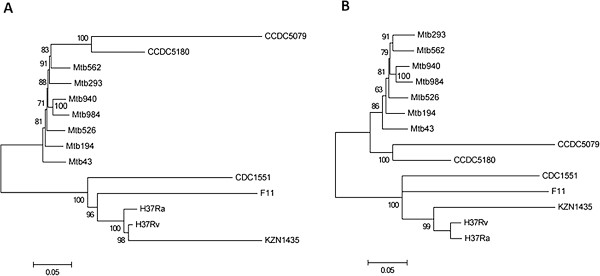
**Phylogenetic relationships of** ***M. tuberculosis*** **isolates based on SNPs from whole genome sequences.** The trees were constructed by the neighbor-joining method **(A)** or maximum-likelihood method **(B)**. Only bootstrap values >50% are shown (n = 1000 replicates).

Two phylogenetic trees including a neighbor-joining (NJ) tree and a maximum-likelihood (ML) tree were created based on SNPs from whole genome sequences of the 7 clinical *M. tuberculosis* isolates and other 7 completely sequenced *M. tuberculosis* strains. The phylogenetic relationships among different clinical isolates were similar in two phylogenetic trees (Figure 2). The 7 newly sequenced Chinese clinical isolates as well as the two previously sequenced Beijing lineage strains CCDC5079 and CCDC5180 formed a single clade.

## Discussion

To determine the genome components and variations within natural populations of *M. tuberculosi*s isolates and to better understand the evolution of drug resistance among those isolates, we explored the feasibility of using deep genome sequencing to characterize variations in clinical *M. tuberculosis* isolates with different drug susceptibility profiles. Our results suggest that the level of genetic diversity is independent of the drug resistance phenotype, since the isolates with different drug resistance profiles harbored similar numbers of SNPs, nucleotide diversity (Л values), and selection intensity (dN/dS values). The relatively high number of SNPs we identified in all isolates could be partially caused by natural variation, as we included all genes from 7 strains isolated from patients diversely located. Selective diversification of *M. tuberculosis* isolates might also explain an association between host response and strain genetic background as previously reported [12, 13]. Several lines of evidence in this study support a significant role of natural selection in shaping *M. tuberculosis* genomes. First, the SNP distribution in genomes is not random, suggesting that diversifying selection is at work notably in certain genes such as those that play a role in cell wall/membrane/envelope biogenesis (class M) and in general function (class R), which tend to accumulate an excess of SNPs [7, 12, 14, 15]. Second, in the SNP density map, many genes located in the regions with significantly high SNP density are involved in host–pathogen interactions and may contribute to strain-specific virulence attributes. For example, one region corresponds to a previously reported virulence operon including the genes Rv0986–Rv0988 that are present in one of horizontal genetic transfer (HGT) regions [7, 16, 17]. Another region with high density of SNPs was found in the ESX-1 locus (RD1 region), which includes a type VII secretion system [18]. But in the absence of the information about strain-specific differences in virulence, the high number of SNPs could also be the result of lateral gene transfer. Third, the dN/dS values were less than 1 for the genomes of all 7 isolates analyzed, consistent with genome-wide purifying selection. We have previously shown that the dN/dS values for coding regions of drug resistance-associated genes in MDR and XDR isolates were higher than 1, suggesting that exposure to drugs is among the major forces driving the high dN/dS ratios in those drug resistance-associated genes [19]. But as suggested in this study, on the genome-wide scale, the clinical *M. tuberculosis* strains with different drug resistance profiles undergo similar levels of purifying selection. Consistently, results from a recent study suggest that the dominant effect of selection on natural *M. tuberculosis* population is removal of novel variants, with exceptions in certain group of genes such as those involved in defense [20].

Indels have a wide range of effects as a very important cause of phenotypic variability. The acquisition and loss of certain genes could provide pathogens with some advantages during infection and transmission. Thus, the Indel loci identified in this study are candidates for drug resistance or virulence-associated factors that may represent evolutionary signatures during the co-evolution of humans and pathogens. For example, the deletion of a polyketide synthase gene (*pks5*) with high homology to mycocerosic acid synthase is particularly intriguing because the product of this gene may be involved in the production of multimethylated branched lipids [21]. In addition, the *pks5* mutant strain of *M. tuberculosis* H37Rv was shown to display severe growth defects in mice [22]. It is also worth noting that sixteen of those Indels spanned PE-PPE-PGRS genes, which have been considered a major source of antigenic variability [10]. In addition, two of those unique proteins code for putative membrane proteins (including MmpL1 and MmpL4) and may directly alter the interactions between pathogens and their hosts [23]. Since we identified many Indels including some of those above-mentioned virulence-associated genes within both drug-susceptible and drug-resistant strains, our results suggest that drug resistance in *M. tuberculosis* is not necessarily an indication of increased virulence. Our findings are consistent with the notion that the virulence of individual clinical *M. tuberculosis* isolate is dependent on multiple factors including strain genetic background and host immune responses [24].

A highly significant inverse correlation between the presence of CRISPR-cas locus and acquired antibiotic resistance was observed in *E. faecalis*, suggesting that antibiotic use inadvertently selects for enterococcal strains with compromised genome defense [25]. But in this study, no functional genes were identified in CRISPR locus and no correlation between antibiotic resistance and the presence of CRISPR-cas locus was observed in clinical *M. tuberculosis* isolates.

Using our previously established method of automatic TBDReaMDB-coupled analysis for drug resistance-associated mutations in *M. tuberculosis* isolates [19], we detected 25 types of unreported mutations, as well as 20 known or putative drug efflux pumps with non-sense SNPs in MDR, pre-XDR and XDR *M. tuberculosis* isolates, but we could not establish the association between over expression of those mutated drug efflux pumps with increased drug resistance in *M. tuberculosis.* It was reported previously that mutations or overexpression of Rv0194 and Rv2686c are associated with increased resistance to multiple drugs in *M. tuberculosis*[26, 27]. But according to another recent study which aimed to compare the differences of the expression of 15 putative multidrug efflux pump genes in clinically isolated drug sensitive and MDR *M. tuberculosis* isolates, all the tested putative multidrug efflux pump genes in the drug-sensitive and MDR *M. tuberculosis* isolates have similar rates of expression [28]. Thus, the existence of mutations and over expression of the efflux pump genes might not be necessarily associated with increased drug resistance.

By closely examining the correlation of the phenotypic drug susceptibility profiles of the strains with mutations identified in their drug resistance-associated genes, we identified a few potential new genetic determinants of drug resistance. For example, while 5 (Mtb194, Mtb293, Mtb940, Mtb984, Mtb43) of the 7 strains exhibited phenotypic resistance to ofloxacin and levofloxacin, only 2 of them (Mtb984 and Mtb43) had *gyrA* D94G mutation known to confer resistance to fluoroquinolones. The other 3 had the same *gyrA* E21Q, G668D, and S95T mutations seen in fluoroquinolone susceptible strain Mtb526, indicating that these mutations are not the source of fluoroquinolone resistance. Similarly, among the 6 strains showing phenotypic resistance to rifampicin, 3 (Mtb194, Mtb293, Mtb940) only had the *rpoB* A1075 mutation, which was also present in the susceptible strain, suggesting the presence of other unknown mechanisms for rifampicin resistance in them. Since we identified no mutations by further examining other drug resistance-associated genes such as *gyrB*, *gidB* and *eis* in those strains [29], we then performed genetic studies for those newly identified potential drug resistance-associated mutations, but failed to confirm their function in causing drug resistance either (data not shown). Thus, our observations demonstrated that though certain drug resistance-associated mutations such as *rpoB* S531L, *katG* S315T, *gyrA* D94G, *embB* M306V, *rpsL* K43R, and *rrs* A1401G could serve as useful markers for rapid detection of resistance in the clinical *M. tuberculosis* isolates, the accuracy and sensitivity of genetic-based drug resistance assays still need to be increased by further elucidation of unknown mechanisms of drug resistance, especially for second-line drugs [29–31]. It should also be pointed out that confirming drug resistance-associated mutations by genetic study could only examine the function of individual gene mutation without taking into consideration the whole genetic background of the strain, while based on the whole genome sequencing studies by us and a few others, there might be no common causes of drug resistance to multiple drugs. Rather, the MDR and XDR phenotypes could result from a combination of mutations in the genomes [15, 32, 33].

The phylogenetic relationships among different clinical isolates were similar in two phylogenetic trees based on whole genome SNPs. The whole genome sequencing has been proposed as a sort of “gold standard” for strain typing in *M. tuberculosis* since it clarifies previous strain typing approaches used for phylogenetic and epidemiologic studies and provides more detailed genomic variation information. The observation that MDR, pre-XDR, and XDR isolates were located sporadically on different branches in phylogenetic trees based on SNPs from whole genome sequences of the 14 *M. tuberculosis* isolates further confirms our previous observation that they have evolved and acquired mutations independently on multiple occasions. The observation that isolates from China were phylogenetically distant from the isolates from other regions such as the KZN strain from South Africa in the phylogenetic trees also confirmed our previous observation that drug-resistant *M. tuberculosis* strains from different geographic regions have distinct evolutionary pathways [19]. The close phylogenetic relatedness among the 7 clinical *M. tuberculosis* isolates could also be best supported by the analysis of specific SNPs in drug resistance-associated genes. The presence of identical uncommon mutations in many of those genes among the 7 strains (e.g. I322V in *Rv1592c*, R463L in *katG*, A1075 in *rpoB*, G668D in *gyrA* etc.) is indicative of a single cluster of strains circulating in the population. The finding of high levels of clustering and minimal strain diversity among MDR/XDR *M. tuberculosis* strains within a population has been described previously [34].

This study has several limitations. Firstly, since the 7 clinical *M. tuberculosis* isolates included in the analysis all belonged to the Beijing lineage, thus it is possible that similarities and differences between different strain groups may be explained by phylogenetic lineages, rather than phenotypic differences. By comparing our 7 clinical isolates with 5 previously described clinical isolates from diverse lineages and countries of origin, we did observe significantly higher Л value for those 5 previously described clinical isolates. However, when we analyzed our 7 Beijing lineage isolates together with two previously described Beijing lineage isolates (CCDC5180: resistant to four first-line drugs; CCDC5079: susceptible strain), the Л value increased significantly, we thus suggest that genomic variations we observed among different groups of isolates are unlikely caused completely by phylogenetic lineages, but rather associated with diverse phenotypes of the isolates. Secondly, this study was limited by the relatively small number of isolates included in the analysis. It is likely that a larger sample with diverse lineages and countries of origin would probably reveal more information on genomic variations and evolution of drug-resistant *M. tuberculosis* strains.

## Conclusions

In this study, by performing whole genome sequencing study, we show that though clinical *M. tuberculosis* isolates have a certain degree of similarity in their genetic make-up, they exhibit distinct variations in terms of the distribution of SNP, Indels, CRISPR-cas locus, as well as the nucleotide diversity and selection intensity. No generalizable differences were identified between drug-susceptible and drug-resistant isolates on the genomic scale. Our study provides evidence strengthening the notion that the evolution of drug resistance among clinical *M. tuberculosis* isolates is clearly a complex and diversified process. Several questions remain further in-depth investigations, such as whether drug susceptibility is affected by the deletion of specific genes and disabling of specific metabolic pathways. In addition, further studies using a larger sampling of *M. tuberculosis* isolates from diverse lineages are warranted to better understand the evolution of drug-resistant *M. tuberculosis* strains.

## Methods

### Selection of strains for genome sequencing and comparative genomic analysis

Seven *M. tuberculosis* clinical strains used for whole genome sequencing in this study were obtained from a TB referral hospital in Beijing, China during the period 2009–2011 [3]. The epidemiologic and clinical data of the patients were extracted from the subjects’ medical records. The selected *M. tuberculosis* clinical isolates had different antibiotic susceptibility profiles (including 1 susceptible isolate, 1 MDR isolate, 3 pre-XDR isolates and 2 XDR isolate). The median age of the 7 patients were 39.02 (range: 21–72) years. All 7 patients were HIV-negative adults. All 7 isolates have the Beijing spoligotype (000000000003771) based on the virtual spoligotyping analysis results. For comparative analysis, genome sequences of two lab strains including H37Rv (NC_000962) and H37Ra (NC_009525) as well as other five previously sequenced clinical isolates including KZN_1435 (NC_012943), F11 (NC_009565), CDC1551 (NC_002755), CCDC5079 (NC_017523), and CCDC5180 (NC_017522) were downloaded from the NCBI website (http://fttp://ftp.ncbi.nih.gov/genomes/Bacteria/). This study was approved by the Ethics Committee of the 309 Hospital and the Institute of Microbiology, Chinese Academy of Sciences, Beijing, China.

### Cultures and drug susceptibility testing

Cultures and drug susceptibility testing (DST) were conducted as described previously [19]. Briefly, sputum specimens were collected, treated and cultured according to the manufacturer’s instructions using the BACTEC MGIT 960 system (Becton Dickinson Diagnostic Systems, Sparks, MD, USA). Cultures positive for growth were examined by microscopy for the presence of acid-fast bacilli after Ziehl-Neelsen staining. Identification of *M. tuberculosis* was performed using p-nitrobenzoic acid and thiophene carboxylic acid hydrazine resistance tests as well as PCR tests. *M. tuberculosis* isolates were further confirmed by 16S rDNA sequencing. DST was conducted using the indirect proportion method on Middlebrook 7H10 agar containing 10% oleic acid-albumin-dextrose-catalase (Difco) and 0.5% glycerol according to the WHO guidelines. The concentrations of the drugs used were as follows: isoniazid (0.2 ug/mL), rifampicin (1 ug/mL), ethambutol (5 ug/mL), streptomycin (2 ug/mL), pyrazinamide (100 ug/mL), ofloxacin (2 ug/mL), levofloxacin (2 ug/mL), kanamycin (5 ug/mL), capreomycin (10 ug/mL), amikacin (1 ug/mL), ethionamide (5 ug/mL), para-aminosalicylic acid (2 ug/mL). Quality control was performed during susceptibility testing using the reference strains provided by the National institute for the control of pharmaceutical and biological products (China). All drugs were obtained from Sigma Life Science Company (USA).

### Genotyping of *M. tuberculosis* isolates

In silico MIRU-VNTR genotyping of the *M. tuberculosis* isolates was conducted. To predict the number of repeats at each locus of MIRUs, 24 VNTR sequences from H37Rv genome were aligned to each assembled genome. The Tandem Repeat Finder algorithm was also used to predict the MIRU-VNTR type of each strain [35]. The in silico MIRU-VNTR results were confirmed by performing experiments following the 24 locus MIRU-VNTR genotyping protocol described by Supply et al. [6]. Virtual spoligotyping was performed by aligning (without gaps) all the reads obtained for each strain against each of the 43 spacer sequences (26-bp oligos) from the direct repeats (DR) regions. The number of matching reads for each spacer was counted, considering both forward and reverse-complemented sequences, and accepting up to 1 nucleotide mismatch. Spacers with 0 matches were interpreted as missing. In addition, we also used SpolPred [36], a well-established genotyping technique based on the presence of unique DNA sequences in *M. tuberculosis*, to predict the spoligotype of each strain.

### DNA preparation and whole genome sequencing

A single colony from 7H10 plate was transferred into 7H9 liquid medium supplemented with OADC and Tween-80, cultured to 0.5 at OD_600_, harvested by centrifugation and resuspended in TE pH8.0 [0.01 M Tris–HCl, 0.001 M EDTA (pH 8.0)]. Genomic DNA was extracted with phenol/chloroform/isoamyl alcohol (25: 24: 1, v/v), precipitated with isopropanol, washed with 75% ethanol and finally resuspended in TE pH8.0. Genome sequencing was performed by BerryGenomics (Beijing, China). We used a whole genome shotgun sequencing strategy and Illumina Genome Analyser sequencing technology. A 100 bp paired-end run was performed with the seven *M. tuberculosis* strains in two lanes. Genomic DNA was sheared by a nebulizer to generate DNA fragments for the Illumina Paried-End Sequencing method. DNA libraries (15–30 ng/μl) were constructed by ligating the specific oligonucleotides (Illumina adapters) designed for PE sequencing to both ends of DNA fragments with the TA cloning method. The ligated DNA was then size selected on a 2% agarose gel. DNA fragments of about 500 bp were excised from the gel. DNA was then recovered using a Qiagen gel extraction kit and was PCR amplified to produce the final DNA library. Five picomoles of DNA from each strain were loaded onto two lanes of the sequencing chip, and the clusters were generated on the cluster generation station of the GAIIx using the Illumina cluster generation kit. Bacteriophage ×174 DNA was used as a control. In the case of paired-end reads, distinct adaptors from Illumina were ligated to each end with PCR primers that allowed reading of each end as separate runs. The sequencing reaction was run for 100 cycles (tagging, imaging, and cleavage of one terminal base at a time), and four images of each tile on the chip were taken in different wavelengths for exciting each base-specific fluorophore. For paired-end reads, data were collected as two sets of matched 100-bp reads. Reads for each of the indexed samples were then separated using a custom Perl script (Additional file 7: Script 1). Image analysis and base calling were done using the Illumina GA Pipeline software.

### Genome assembly and annotation

Short reads were assembled using SOAPdenovo (http://soap.genomics.org.cn), a genome assembler developed specifically for next-generation short-read sequences. As the algorithm is sensitive to sequencing errors, low-quality reads were filtered, and high-quality reads were used for *de novo* assembly. Sequences were filtered for low quality reads using the DynamicTrim and LengthSort Perl scripts within SolexaQA. These scripts trimmed each read to the longest contiguous read segment for which the quality score at each base was greater than p = 0.05 (approximately equivalent to a Phred score of 13), and then removed sequence reads shorter than 25 bp respectively. Where one sequence of a pair was removed, the remaining sequence was put into a separate file and used as a singleton during *de novo* assembly. The SOAP GapCloser was also used to close gaps where possible after assembly.

The protein-coding genes were predicted using Glimmer 3.02 [37], while tRNAscan-SE [38] and RNAmmer [39] were used to identify tRNA and rRNA, respectively. The genome sequence was also uploaded into Rapid Annotation using Subsystem Technology (RAST) [40] to check the annotated sequences. The functions of predicted protein-coding genes were then annotated through comparisons with the databases of NCBI-NR, COG, and KEGG.

### Nucleotide sequence accession numbers

Whole genome sequencing projects for 7 clinical *M. tuberculosis* isolates Mtb562, Mtb194, Mtb293, Mtb526, Mtb940, Mtb984, and Mtb43 have been deposited in GenBank under accession numbers AUTG00000000, AUNH00000000, AUPX00000000, AUTF00000000, AUTX00000000, AUTY00000000, and AUPO00000000, respectively.

### SNP detection and analysis

For the sequenced genomes, SOAPsnp (http://soap.genomics.org.cn/soapsnp.html) was used to score SNPs from aligned reads [41]. The short reads were aligned onto the H37Rv genome reference using the SOAP2 program [18]. To obtain reliable alignment hits, at most two mismatches were allowed between the read and the reference. The alignments with the least number of differences were defined as “best hits.” If there was only one single best hit for a read, then the read was taken as uniquely placed; a read with multiple equal best hits was taken as repeatedly placed. For paired-end reads, two reads belonging to a pair were aligned together with both in the correct orientation and with a proper span size on the reference. The 100-bp reads that were generated for each strain were mapped against H37Rv as a reference sequence via ungapped alignments allowing up to two mismatches. For reads that mapped to multiple locations, one was chosen at random. For paired-end data, mapping locations of each read were restricted to sites within 300 bp of mapping locations of its partner. SOAPsnp results were filtered as follows: 1) The read coverage of the SNP site was more than five; 2) The Illumina quality score of either allele was more than 30; 3) The count of all mapped best base is more than two times the count of all mapped second best base. In addition, BWA 0.6.2 [42] and SAMtools 0.1.18 [43] were used to confirm our results. The Illumina reads were first aligned by BWA with default parameters for each sample. The aligned results were piped to SAMtools for conversion of BWA output format to BAM format and to perform SNP analysis. For the other genomes, all specific SNPs for each strain were manually inspected by taking into account if SNPs were detected by the two aligners including MAUVE [44] and MUMmer 3.2 [45]. From all SNPs identified in the sequenced genome sequences, the density of SNPs was calculated throughout the *M. tuberculosis* H37Rv genome using a sliding-window size of 5 kb (step of the sliding window = 5 kb). This analysis led to the construction of a SNP clustering map using Circos [46].

### Insertion and deletion (Indel) analysis

Three different methods were used to detect Indels: 1) Multiple alignment of genomic sequences was performed by using Mauve multiple alignment software and the progressive alignment option. The output file produced by Mauve was parsed by using a custom Perl script to retrieve multiple aligned sequences for Indel loci (Additional file 8: Script 2); 2) For each genome-wide Ilumina sequence dataset, the sequence reads were aligned against the reference genome sequence using BWA 0.6.2 [42]. Then SAMTOOLS 0.1.18 [43], which is based on a Bayesian model for Indel calling, was used to perform the analysis using the default Indel detection parameters, with a small increase in the coverage threshold (-D 200); 3) Indel from paired-end mapping data were identified and visualized with inGAP-SV [47], which uses read depth and read pair data to detect and visualize large and complex sequence variation.

### CRISPR locus identification

For published genome sequences, CRISPR loci were retrieved from the CRISPRdb database [48]. Alternatively, the detection of CRISPR loci in our 7 draft genome sequences was achieved using CRISPRFinder [48]. BLAST was used for similarity searches between CRISPR spacer sequences and existing sequences in the GenBank database limited to Bacteria (taxid: 2) or Viruses (taxid: 10239) entries. Only matches showing 100% identity over the complete CRISPR spacer sequences were retained, and matches to sequences found within CRISPR loci were ignored.

### Identification of gene mutations associated with drug resistance

Mutations in *M. tuberculosis* antibiotic resistance-associated genes and inter-genic regions were downloaded from the TB Drug Resistance Mutation Database (TBDReaMDB) [49], a comprehensive database providing all reported mutations associated with TB drug resistance through a publicly accessible web site: http://www.tbdreamdb.com, to provide information for comparison analysis of drug resistance-associated mutation profiles for the 7 sequenced *M. tuberculosis* isolates. To confirm the association between specific gene mutations and drug resistance, we amplified the putative drug resistance-associated genes with mutations from the genomic DNA of clinical *M. tuberculosis* isolates by PCR, and cloned them into the plasmid pMV261, a mycobacterial replicating vector, then electroporated the recombinant vectors into the drug-susceptible reference H37Rv strain for drug susceptibility testing. All the experiments were repeated at least 3 times.

### Genetic diversity and selection intensity analysis

The program DnaSP software version 5.10 was used to investigate the genetic diversity of the whole genome sequences of the *M. tuberculosis* isolates [50]. The genetic diversity were measured by haplotype (H), diversity of haplotype (Hd), nucleotide diversity (p), and the average number of nucleotide differences (K). The sequences of the coding regions from each isolate were concatenated and the resulting sequences were used to determine the number of non-synonymous (dN) and synonymous (dS) substitutions per site. To test the selection intensity, the ratios of dN/dS were calculated for each pairwise comparison, and two-sided Z-test was used to determine the level of significance.

### Phylogenetic analysis

The neighbor-joining (NJ) and maximum-likelihood (ML) phylogenetic trees were constructed in MEGA5 [51] based on SNPs from whole genome sequences. The reliability of each node was estimated from 1000 random bootstrap resamplings of the data. The phylogenetic data have been deposited in TreeBase under the accession number 15638 (http://purl.org/phylo/treebase/phylows/study/TB2:S15638).

## Electronic supplementary material

Additional file 1: Table S1: Sequencing statistics of *M. tuberculosis* isolates. (DOC 46 KB)

Additional file 2: Table S2: Regions with significantly high SNP density. (DOC 54 KB)

Additional file 3: Figure S1: Distribution of SNPs according to the Clusters of Orthologous Groups (COG) classification. (U) Intracellular trafficking and secretion; (V) Defense mechanisms; (D) Cell cycle control, mitosis, and meiosis; (F) Nucleotide transport and metabolism; (O) Post-translational modification, protein turnover, chaperones; [O] Posttranslational modification, protein turnover, chaperones; [J] Translation, ribosomal structure and biogenesis; (H) Coenzyme transport and metabolism; [M] Cell wall/membrane/envelope biogenesis; [S] Function unknown; [K] Transcription; (P) Inorganic ion transport and metabolism; (T) Signal transduction mechanisms; (G) Carbohydrate transport and metabolism; (N) Cell motility; (C) Energy production and conversion; (L) Replication, recombination, and repair; [E] Amino acid transport and metabolism; (I) Lipid transport and metabolism; (R) General function;. (Q) Secondary metabolites biosynthesis, transport, and catabolism. (*) Class with significant over-representation and less-representation of SNPs (p < 0.01). (TIFF 2 MB)

Additional file 4: Figure S2: Overview of the CRISPR loci in *M. tuberculosis* strains. Spacers are shown as diamonds and repeats as rectangles. In each CRISPR, spacers with identical sequence in the studied genomes are shown in the same color. (TIFF 4 MB)

Additional file 5: Table S3: Known or putative drug efflux pumps with non-synonymous SNPs in MDR, pre-XDR and XDR *M. tuberculosis* isolates but not in H37Rv strain. (DOC 53 KB)

Additional file 6: Table S4: DNA diversity and selection intensity analysis for the whole genome sequences of *M. tuberculosis* isolates. (DOC 55 KB)

Additional file 7: **Script 1.** The custom Perl script used to separate each of the indexed samples from raw reads. (ZIP 574 bytes)

Additional file 8: **Script 2.** The custom Perl script used to retrieve Indel loci from output file (.xmfa) produced by Mauve. (ZIP 1 KB)
