# Supplementary material for: Comparative genomic analysis of Mycobacterium tuberculosis clinical isolates
Source: BMC Genomics. 2014 Jun 13;15(1):469. doi: 10.1186/1471-2164-15-469 (PMC4070564; doi:10.1186/1471-2164-15-469)
Supplement: Supplementary file 1 — Additional file 1: Table S1: Sequencing statistics of M. tuberculosis isolates. (DOC 46 KB) [file 12864_2013_6147_MOESM1_ESM.doc]

**Table S1 Sequencing statistics of *M. tuberculosis*** isolates

| **Isolates** | **Total raw Pairs** | **Completion (%)** | **Full length (bp)** | **Coverage (**×**)** | **SNPs*** | **Indels*** |
| --- | --- | --- | --- | --- | --- | --- |
| Mtb562 | 12,078,900 | 97.82 | 4298163 | 490 × | 1428 | 101 |
| Mtb526 | 10,055,817 | 97.53 | 4302478 | 370 × | 1409 | 85 |
| Mtb194 | 9,474,941 | 97.67 | 4315524 | 400 × | 1416 | 58 |
| Mtb293 | 9,358,295 | 97.61 | 4310427 | 390 × | 1437 | 71 |
| Mtb940 | 12,195,654 | 97.66 | 4306144 | 510× | 1422 | 56 |
| Mtb984 | 13,475,588 | 97.71 | 4308572 | 560× | 1464 | 87 |
| Mtb43 | 4,889,739 | 97.43 | 4308328 | 200 × | 1409 | 96 |

*****The number of SNPs and Indels are assessed relative to H37Rv.
