# Supplementary material for: Comparative genomic analysis of Mycobacterium tuberculosis clinical isolates
Source: BMC Genomics. 2014 Jun 13;15(1):469. doi: 10.1186/1471-2164-15-469 (PMC4070564; doi:10.1186/1471-2164-15-469)
Supplement: Supplementary file 2 — Additional file 2: Table S2: Regions with significantly high SNP density. (DOC 54 KB) [file 12864_2013_6147_MOESM2_ESM.doc]

**Table S2 Regions with significantly high SNP density.** Note: SNP clustering positions were shown according to H37Rv genome sequence

| **Region of SNPs clustering** | **Genes/Products** | **Numbers of non-repetitive nsSNPs in the designated regions (isolate: numbers of nsSNPs)** | **Numbers of non-repetitive sSNPs in the designated regions (isolate: numbers of sSNPs)** |
| --- | --- | --- | --- |
| 2865000-2869999 | Rv2545:CONSERVED HYPOTHETICAL PROTEIN  lppB:PROBABLE CONSERVED LIPOPROTEIN LPPB  lppA:PROBABLE CONSERVED LIPOPROTEIN LPPA  Rv2542:CONSERVED HYPOTHETICAL PROTEIN | 9(Mtb562:8,Mtb194:8,Mtb293:8,Mtb526:8,Mtb940:8,Mtb984:8,Mtb43:8) | 5(Mtb562:5,Mtb194:5,Mtb293:5,Mtb526:5,Mtb940:5,Mtb984:5,Mtb43:5) |
| 3730000-3734999 | PPE54:PPE FAMILY PROTEIN | 5(Mtb562:3,Mtb194:4,Mtb293:1,Mtb526:2,Mtb940:2,Mtb984:3,Mtb43:3) | 6(Mtb562:3,Mtb194:1,Mtb293:2,Mtb526:4,Mtb940:1,Mtb984:3,Mtb43:5) |
| 35000-39999 | bioF2:POSSIBLE 8-AMINO-7-OXONONANOATE SYNTHASE BIOF2 (AONS) (8-AMINO-7-KETOPELARGONATE SYNTHASE)  fadD34:PROBABLE FATTY-ACID-CoA LIGASE FADD34 (FATTY-ACID-CoA SYNTHETASE) (FATTY-ACID-CoA SYNTHASE)  Rv0036c:CONSERVED HYPOTHETICAL PROTEIN  Rv0034:CONSERVED HYPOTHETICAL PROTEIN | 5(Mtb562:3,Mtb194:3,Mtb293:4,Mtb526:3,Mtb940:3,Mtb984:4,Mtb43:3) | 5(Mtb562:3,Mtb194:3,Mtb293:4,Mtb526:3,Mtb940:4,Mtb984:4,Mtb43:3) |
| 3880000-3884999 | Rv3466:CONSERVED HYPOTHETICAL PROTEIN  Rv3467:CONSERVED HYPOTHETICAL PROTEIN  rmlC:dTDP-4-DEHYDRORHAMNOSE 3,5-EPIMERASE RMLC (dTDP-4-KETO-6-DEOXYGLUCOSE 3,5-EPIMERASE) | 8(Mtb562:3,Mtb194:3,Mtb293:3,Mtb526:6,Mtb940:3,Mtb984:3,Mtb43:5) | 2(Mtb562:1,Mtb194:1,Mtb293:1,Mtb526:1,Mtb940:1,Mtb984:1,Mtb43:2) |
| 3135000-3139999 | Rv2828c:CONSERVED HYPOTHETICAL PROTEIN Rv2830c:CONSERVED HYPOTHETICAL PROTEIN  ugpC:PROBABLE Sn-GLYCEROL-3-PHOSPHATE TRANSPORT ATP-BINDING PROTEIN ABC TRANSPORTER UGPC  ugpB:PROBABLE Sn-GLYCEROL-3-PHOSPHATE-BINDING LIPOPROTEIN UGPB  echA16:PROBABLE ENOYL-CoA HYDRATASE ECHA16 (ENOYL HYDRASE) (UNSATURATED ACYL-CoA HYDRATASE) (CROTONASE) | 5(Mtb562:3,Mtb194:3,Mtb293:3,Mtb526:5,Mtb940:3,Mtb984:3,Mtb43:2) | 4(Mtb562:1,Mtb194:1,Mtb293:1,Mtb526:2,Mtb940:1,Mtb984:1,Mtb43:2) |
| 3750000-3754999 | PPE55:PPE FAMILY PROTEIN  Rv3349c:PROBABLE TRANSPOSASE | 5(Mtb562:5,Mtb194:5,Mtb293:5,Mtb526:5,Mtb940:5,Mtb984:5,Mtb43:5) | 4(Mtb562:4,Mtb194:4,Mtb293:4,Mtb526:4,Mtb940:4,Mtb984:4,Mtb43:4) |
| 1275000-1279999 | Rv1148c:CONSERVED HYPOTHETICAL PROTEIN  Rv1152:PROBABLE TRANSCRIPTIONAL REGULATORY PROTEIN | 3(Mtb562:0,Mtb194:1,Mtb293:0,Mtb526:0,Mtb940:0,Mtb984:0,Mtb43:3) | 5(Mtb562:4,Mtb194:3,Mtb293:2,Mtb526:2,Mtb940:4,Mtb984:4,Mtb43:2) |
| 2335000-2339999 | Rv2082:CONSERVED HYPOTHETICAL PROTEIN  Rv2079:CONSERVED HYPOTHETICAL PROTEIN  Rv2078:HYPOTHETICAL PROTEIN  lppJ:Possible lipoprotein lppJ | 7(Mtb562:6,Mtb194:5,Mtb293:5,Mtb526:5,Mtb940:5,Mtb984:6,Mtb43:6) | 1(Mtb562:1,Mtb194:1,Mtb293:1,Mtb526:1,Mtb940:1,Mtb984:1,Mtb43:1) |
| 2625000-2629999 | esxP:PUTATIVE ESAT-6 LIKE PROTEIN ESXP (ESAT-6 LIKE PROTEIN 7) plcC:PROBABLE PHOSPHOLIPASE C 3 PLCC  esxO:PUTATIVE ESAT-6 LIKE PROTEIN ESXO (ESAT-6 LIKE PROTEIN 6) | 3(Mtb562:3,Mtb194:3,Mtb293:3,Mtb526:3,Mtb940:3,Mtb984:3,Mtb43:3) | 5(Mtb562:5,Mtb194:4,Mtb293:5,Mtb526:5,Mtb940:5,Mtb984:5,Mtb43:3) |
| 15000-19999 | pknA:TRANSMEMBRANE SERINE/THREONINE-PROTEIN KINASE A PKNA (PROTEIN KINASE A) (STPK A)  trpG:POSSIBLE ANTHRANILATE SYNTHASE COMPONENT II TRPG (GLUTAMINE AMIDOTRANSFERASE)  pknB:TRANSMEMBRANE SERINE/THREONINE-PROTEIN KINASE B PKNB (PROTEIN KINASE B) (STPK B)  pbpA:PROBABLE PENICILLIN-BINDING PROTEIN PBPA | 6(Mtb562:4,Mtb194:4,Mtb293:5,Mtb526:4,Mtb940:5,Mtb984:4,Mtb43:4) | 1(Mtb562:0,Mtb194:1,Mtb293:0,Mtb526:0,Mtb940:0,Mtb984:0,Mtb43:1) |
| 260000-264999 | Rv0219:PROBABLE CONSERVED TRANSMEMBRANE PROTEIN Rv0218:PROBABLE CONSERVED TRANSMEMBRANE PROTEIN Rv0221:CONSERVED HYPOTHETICAL PROTEIN  lipW:POSSIBLE ESTERASE LIPW | 5(Mtb562:2,Mtb194:4,Mtb293:3,Mtb526:2,Mtb940:2,Mtb984:2,Mtb43:2) | 2(Mtb562:1,Mtb194:1,Mtb293:1,Mtb526:1,Mtb940:1,Mtb984:2,Mtb43:1) |
| 335000-339999 | PE_PGRS3:PE-PGRS FAMILY PROTEIN  PE_PGRS4:PE-PGRS FAMILY PROTEIN | 2(Mtb562:2,Mtb194:2,Mtb293:2,Mtb526:0,Mtb940:1,Mtb984:2,Mtb43:2) | 5(Mtb562:2,Mtb194:1,Mtb293:0,Mtb526:4,Mtb940:2,Mtb984:1,Mtb43:1) |
| 1095000-1099999 | PE_PGRS18:PE-PGRS FAMILY PROTEIN  mprB:PROBABLE TWO COMPONENT SENSOR KINASE MPRB  mprA:MYCOBACTERIAL PERSISTENCE REGULATOR MRPA (TWO COMPONENT RESPONSE TRANSCRIPTIONAL REGULATORY PROTEIN) | 3(Mtb562:3,Mtb194:3,Mtb293:3,Mtb526:3,Mtb940:3,Mtb984:3,Mtb43:2) | 4(Mtb562:4,Mtb194:4,Mtb293:4,Mtb526:4,Mtb940:4,Mtb984:4,Mtb43:4) |
| 1100000-1104999 | Rv0987:PROBABLE ADHESION COMPONENT TRANSPORT TRANSMEMBRANE PROTEIN ABC TRANSPORTER  Rv0986:PROBABLE ADHESION COMPONENT TRANSPORT ATP-BINDING PROTEIN ABC TRANSPORTER  pepD:PROBABLE SERINE PROTEASE PEPD (SERINE PROTEINASE) (MTB32B) | 4(Mtb562:3,Mtb194:3,Mtb293:3,Mtb526:3,Mtb940:3,Mtb984:3,Mtb43:4) | 3(Mtb562:3,Mtb194:3,Mtb293:3,Mtb526:3,Mtb940:3,Mtb984:3,Mtb43:3) |
| 1105000-1109999 | Rv0992c:CONSERVED HYPOTHETICAL PROTEIN  galU:PROBABLE UTP--GLUCOSE-1-PHOSPHATE URIDYLYLTRANSFERASE GALU (UDP-GLUCOSE PYROPHOSPHORYLASE) (UDPGP)  Rv0987:PROBABLE ADHESION COMPONENT TRANSPORT TRANSMEMBRANE PROTEIN ABC TRANSPORTER  grcC2:PROBABLE POLYPRENYL-DIPHOSPHATE SYNTHASE GRCC2 (POLYPRENYL PYROPHOSPHATE SYNTHETASE)  Rv0988:POSSIBLE CONSERVED EXPORTED PROTEIN Rv0990c:HYPOTHETICAL PROTEIN | 7(Mtb562:6,Mtb194:5,Mtb293:5,Mtb526:5,Mtb940:6,Mtb984:5,Mtb43:5) | 0(Mtb562:,Mtb194:,Mtb293:,Mtb526:,Mtb940:,Mtb984:,Mtb43:) |
| 1955000-1959999 | Rv1729c:CONSERVED HYPOTHETICAL PROTEIN  Rv1730c:POSSIBLE PENICILLIN-BINDING PROTEIN | 6(Mtb562:2,Mtb194:1,Mtb293:3,Mtb526:1,Mtb940:2,Mtb984:2,Mtb43:1) | 1(Mtb562:0,Mtb194:0,Mtb293:0,Mtb526:0,Mtb940:1,Mtb984:0,Mtb43:0) |
| 2160000-2164999 | aceAa:PROBABLE ISOCITRATE LYASE aceAa [FIRST PART] (ISOCITRASE) (ISOCITRATASE) (ICL)  PPE34:PPE FAMILY PROTEIN | 6(Mtb562:2,Mtb194:2,Mtb293:1,Mtb526:2,Mtb940:2,Mtb984:3,Mtb43:2) | 1(Mtb562:1,Mtb194:1,Mtb293:1,Mtb526:1,Mtb940:1,Mtb984:1,Mtb43:1) |
| 2300000-2304999 | pks12:Probable polyketide synthase pks12 | 3(Mtb562:2,Mtb194:2,Mtb293:2,Mtb526:3,Mtb940:2,Mtb984:2,Mtb43:1) | 4(Mtb562:3,Mtb194:3,Mtb293:3,Mtb526:4,Mtb940:3,Mtb984:3,Mtb43:2) |
| 3245000-3249999 | ppsA:PHENOLPTHIOCEROL SYNTHESIS TYPE-I POLYKETIDE SYNTHASE PPSA | 4(Mtb562:3,Mtb194:4,Mtb293:4,Mtb526:4,Mtb940:4,Mtb984:4,Mtb43:3) | 3(Mtb562:2,Mtb194:2,Mtb293:3,Mtb526:3,Mtb940:3,Mtb984:2,Mtb43:2) |
| 3840000-3844999 | PPE57:PPE FAMILY PROTEIN Rv3424c:HYPOTHETICAL PROTEIN  Rv3428c:POSSIBLE TRANSPOSASE | 6(Mtb562:3,Mtb194:5,Mtb293:3,Mtb526:3,Mtb940:3,Mtb984:4,Mtb43:3) | 1(Mtb562:1,Mtb194:1,Mtb293:1,Mtb526:1,Mtb940:1,Mtb984:1,Mtb43:1) |
| 4215000-4219999 | Rv3770c:HYPOTHETICAL LEUCINE RICH PROTEIN  hisC2:PROBABLE HISTIDINOL-PHOSPHATE AMINOTRANSFERASE HISC2 (IMIDAZOLE ACETOL-PHOSPHATE TRANSAMINASE)  echA21:POSSIBLE ENOYL-CoA HYDRATASE ECHA21 (ENOYL HYDRASE) (UNSATURATED ACYL-CoA HYDRATASE) (CROTONASE)  Rv3773c:CONSERVED HYPOTHETICAL PROTEIN | 5(Mtb562:4,Mtb194:4,Mtb293:4,Mtb526:3,Mtb940:3,Mtb984:3,Mtb43:4) | 2(Mtb562:1,Mtb194:1,Mtb293:1,Mtb526:1,Mtb940:1,Mtb984:1,Mtb43:2) |
| 4220000-4224999 | Rv3777:PROBABLE OXIDOREDUCTASE  Rv3776:CONSERVED HYPOTHETICAL PROTEIN | 5(Mtb562:3,Mtb194:3,Mtb293:3,Mtb526:3,Mtb940:4,Mtb984:4,Mtb43:4) | 2(Mtb562:2,Mtb194:2,Mtb293:2,Mtb526:2,Mtb940:2,Mtb984:2,Mtb43:2) |
| 4230000-4234999 | Rv3785:HYPOTHETICAL PROTEIN  Rv3787c:CONSERVED HYPOTHETICAL PROTEIN  Rv3784:POSSIBLE dTDP-GLUCOSE 4,6-DEHYDRATASE  Rv3788:HYPOTHETICAL PROTEIN | 5(Mtb562:3,Mtb194:2,Mtb293:2,Mtb526:1,Mtb940:2,Mtb984:3,Mtb43:1) | 2(Mtb562:2,Mtb194:1,Mtb293:1,Mtb526:1,Mtb940:1,Mtb984:1,Mtb43:1) |
| 4250000-4254999 | accD4:PROBABLE PROPIONYL-CoA CARBOXYLASE BETA CHAIN 4 ACCD4 (PCCASE) (PROPANOYL-COA:CARBON DIOXIDE LIGASE)  fadE35:PROBABLE ACYL-CoA DEHYDROGENASE FADE35  Rv3798:PROBABLE TRANSPOSASE | 3(Mtb562:2,Mtb194:3,Mtb293:2,Mtb526:2,Mtb940:2,Mtb984:2,Mtb43:2) | 4(Mtb562:4,Mtb194:4,Mtb293:4,Mtb526:4,Mtb940:4,Mtb984:4,Mtb43:3) |
| 4355000-4359999 | Rv3879c:HYPOTHETICAL ALANINE AND PROLINE RICH PROTEIN Rv3877:PROBABLE CONSERVED TRANSMEMBRANE PROTEIN | 5(Mtb562:3,Mtb194:3,Mtb293:3,Mtb526:3,Mtb940:3,Mtb984:5,Mtb43:3) | 2(Mtb562:2,Mtb194:2,Mtb293:2,Mtb526:2,Mtb940:2,Mtb984:2,Mtb43:2) |
