# Supplementary figures and images for: Comparative genomic analysis of Mycobacterium tuberculosis clinical isolates
Source: BMC Genomics. 2014 Jun 13;15(1):469. doi: 10.1186/1471-2164-15-469 (PMC4070564; doi:10.1186/1471-2164-15-469)

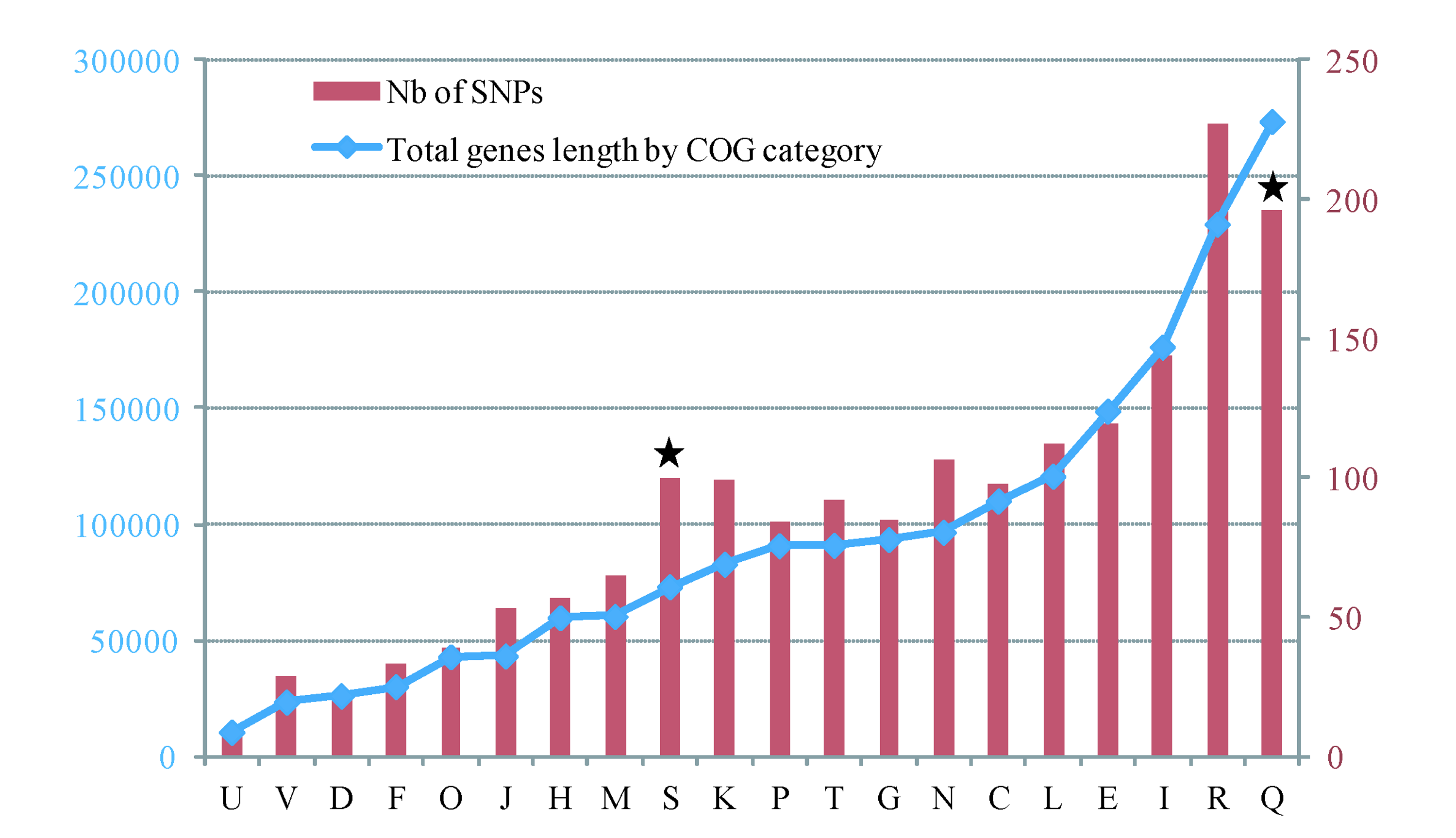

Supplement: Supplementary file 3 — Additional file 3: Figure S1: Distribution of SNPs according to the Clusters of Orthologous Groups (COG) classification. (U) Intracellular trafficking and secretion; (V) Defense mechanisms; (D) Cell cycle control, mitosis, and meiosis; (F) Nucleotide transport and metabolism; (O) Post-translational modification, protein turnover, chaperones; [O] Posttranslational modification, protein turnover, chaperones; [J] Translation, ribosomal structure and biogenesis; (H) Coenzyme transport and metabolism; [M] Cell wall/membrane/envelope biogenesis; [S] Function unknown; [K] Transcription; (P) Inorganic ion transport and metabolism; (T) Signal transduction mechanisms; (G) Carbohydrate transport and metabolism; (N) Cell motility; (C) Energy production and conversion; (L) Replication, recombination, and repair; [E] Amino acid transport and metabolism; (I) Lipid transport and metabolism; (R) General function;. (Q) Secondary metabolites biosynthesis, transport, and catabolism. (*) Class with significant over-representation and less-representation of SNPs (p < 0.01). (TIFF 2 MB) [file 12864_2013_6147_MOESM3_ESM.tiff]

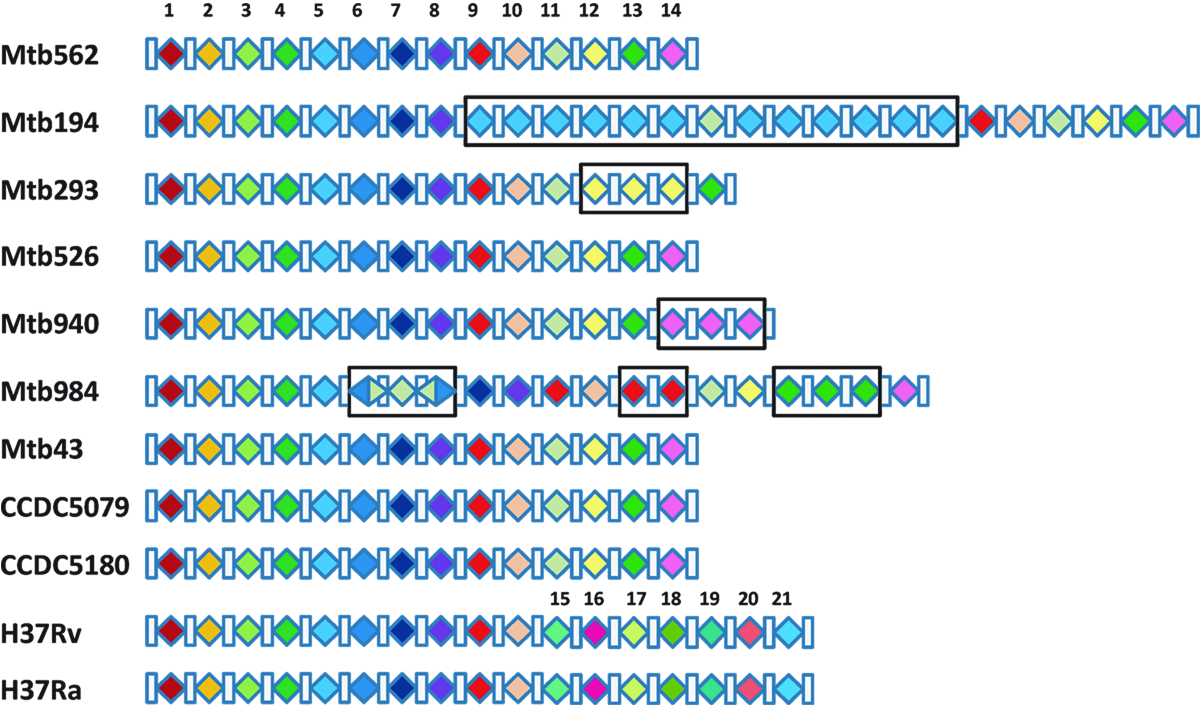

Supplement: Supplementary file 4 — Additional file 4: Figure S2: Overview of the CRISPR loci in M. tuberculosis strains. Spacers are shown as diamonds and repeats as rectangles. In each CRISPR, spacers with identical sequence in the studied genomes are shown in the same color. (TIFF 4 MB) [file 12864_2013_6147_MOESM4_ESM.tiff]
