# Supplementary material for: Comparative genomic analysis of Mycobacterium tuberculosis clinical isolates
Source: BMC Genomics. 2014 Jun 13;15(1):469. doi: 10.1186/1471-2164-15-469 (PMC4070564; doi:10.1186/1471-2164-15-469)
Supplement: Supplementary file 5 — Additional file 5: Table S3: Known or putative drug efflux pumps with non-synonymous SNPs in MDR, pre-XDR and XDR M. tuberculosis isolates but not in H37Rv strain. (DOC 53 KB) [file 12864_2013_6147_MOESM5_ESM.doc]

**Table S3 Known or putative drug efflux pumps with non-synonymous SNPs in MDR, XDR and TDR *M. tuberculosis* isolates but not** in H37Rv strain

| **Rv No.** | **Gene** | **Function** | **Base mutation** | **AA mutation** |
| --- | --- | --- | --- | --- |
| Rv0050 | *ponA1* | Bifunctional penicillin-binding protein 1A/1B | C1891T | P631S |
| Rv0103c | *ctpB* | Cation-transporter P-type ATPase A/B | A65G | L22S |
| Rv0174 | *mce1F* | MCE-family protein MCE1F, putative ABC transport system substrate-binding protein | T1109C | L370P |
| Rv0194 |  | Drugs-transport transmembrane ATP-binding protein ABC transporter | T221C | M74T |
| Rv0425c | *ctpH* | Metal cation transporter P-type ATPase CtpH | T2065C | M689V |
| Rv0507 | *mmpL2* | Transmembrane transport protein MmpL2, putative drug exporter of the RND superfamily | G1277A | R426H |
| Rv0545c | *pitA* | Inorganic phosphate transporter, PiT family | G145A | P49S |
| Rv0589 | *mce2A* | MCE-family protein MCE2A, putative ABC transport system substrate-binding protein | T152C | F51S |
| Rv0676c | *mmpL5* | Transmembrane transport protein MmpL5, putative drug exporter of the RND superfamily | T2842C | I948V |
| Rv3331 | *sugI* | Sugar-transport integral membrane protein SugI | C1268T | P423L |
| Rv3522 | *itp4* | Lipid-transfer protein | C971T | T324I |
| Rv3833 | *araC* | AraC family transcriptional regulator | G313A | V105I |
| Rv0107c | *ctpI* | Cation-transporter ATPase I | G4712A | S1571L |
| Rv0587 | *yrbE2A* | Putative ABC transport system permease protein | C575T | T192I |
| Rv1183 | *mmpL10* | Transmembrane transport protein MmpL10, putative drug exporter of the RND superfamily | G2070A | M690I |
| Rv1200 |  | Integral membrane transport protein | G377A | G126D |
| Rv1730c |  | Penicillin-binding protein | A1361G | M454T |
| Rv1858 | *modB* | Molbdenum-transport integral membrane protein ABC transporter | C57G | I19M |
| Rv2320c | *rocE* | Cationic amino acid transport integral membrane protein RocE | T1328C | H443R |
| Rv2686c |  | Antibiotic ABC transporter transmembrane protein | G626C | P209R |
