# Supplementary material for: Comparative genomic analysis of Mycobacterium tuberculosis clinical isolates
Source: BMC Genomics. 2014 Jun 13;15(1):469. doi: 10.1186/1471-2164-15-469 (PMC4070564; doi:10.1186/1471-2164-15-469)
Supplement: Supplementary file 6 — Additional file 6: Table S4: DNA diversity and selection intensity analysis for the whole genome sequences of M. tuberculosis isolates. (DOC 55 KB) [file 12864_2013_6147_MOESM6_ESM.doc]

**Table S4 DNA diversity and selection intensity analysis for the whole genome sequences of *M. tuberculosis*** isolates

| **Isolates** | **No. of isolates** | **Haplotype** | **Diversity of haplotype** | **Л^a^** | **K^b^** | **dN/dS^c^** | ***P* value by Z-test** |
| --- | --- | --- | --- | --- | --- | --- | --- |
| Mtb562 ^d^ | 2 | 2 | 1.000 | 0.00033 | 1319.0 | 0.608885 | - |
| Mtb526 ^d^ | 2 | 2 | 1.000 | 0.00034 | 1381.0 | 0.637365 | - |
| Mtb194 ^d^ | 2 | 2 | 1.000 | 0.00035 | 1393.0 | 0.620566 | - |
| Mtb293 ^d^ | 2 | 2 | 1.000 | 0.00036 | 1429.0 | 0.629613 | - |
| Mtb940 ^d^ | 2 | 2 | 1.000 | 0.00035 | 1399.0 | 0.616244 | - |
| Mtb984 ^d^ | 2 | 2 | 1.000 | 0.00036 | 1429.0 | 0.61558 | - |
| Mtb43 ^d^ | 2 | 2 | 1.000 | 0.00035 | 1403.0 | 0.628597 | - |
| Drug susceptible isolates | 3 (H37Ra, H37Rv, 562) | 3 | 1.000 | 0.00024 | 955.3 | 0.66891 | - |
| Drug-resistant isolates | 6+ (5180+KZN1435) | 8 | 1.000 | 0.00021 | 849.3 | 0.687259 | <0.001 |
| Lab strains | 2 (H37Ra, H37Rv) | 2 | 1.000 | 0.00004 | 180.0 | 0.765664 | - |
| Clinical strains | 7+5 | 12 | 1.000 | 0.00033 | 1335.7 | 0.66018 | <0.001 |
| 5 ref clinical strains | 5 | 5 | 1.000 | 0.00057 | 2283.1 | 0.6352 | <0.001 |
| 7 our clinical strains | 7 | 7 | 1.000 | 0.00008 | 333.2 | 0.7035 | <0.001 |
| 9 clinical strains | 7+(5180+5079) | 9 | 1.000 | 0.00028 | 736.8 | 0.6617 | <0.001 |

^a^ nucleotide diversity.

^b^ average number of nucleotide differences.

^c^ the rates of non-synonymous and synonymous changes.

^d^ H37Rv was used in pairwise comparisons.
